# Retinoblastoma-binding Protein 9 Suppresses Intestinal Inflammation and Inflammation-induced Tumorigenesis in Mice

**DOI:** 10.1016/j.jcmgh.2024.101435

**Published:** 2024-12-02

**Authors:** Kensuke Hamada, Yuki Nakanishi, Yu Muta, Mayuki Omatsu, Kosuke Iwane, Munehiro Ikeda, Jiayu Chen, Yoko Masui, Naoki Aoyama, Nobukazu Agatsuma, Go Yamakawa, Takahiro Utsumi, Hiroki Kitamoto, Makoto Okabe, Yoshiro Itatani, Takumi Adachi, Koubun Yasuda, Shuji Yamamoto, Akihisa Fukuda, Etsushi Kuroda, Masaki Ohmuraya, Kazutaka Obama, Seiichi Hirota, Hiroki Ikeuchi, Kenji Nakanishi, Hiroshi Seno

**Affiliations:** 1 Department of Gastroenterology and Hepatology, Kyoto University Graduate School of Medicine, Kyoto, Japan; 2 Department of Gastrointestinal Surgery, Kyoto University Graduate School of Medicine, Kyoto, Japan; 3 Department of Immunology, School of Medicine, Hyogo Medical University, Nishinomiya, Japan; 4 Department of Genetics, School of Medicine, Hyogo Medical University, Nishinomiya, Japan; 5 Department of Surgical Pathology, School of Medicine, Hyogo Medical University, Nishinomiya, Japan; 6 Department of Gastroenterological Surgery, School of Medicine, Hyogo Medical University, Nishinomiya, Japan

**Keywords:** Colitis-associated Cancer, Interferon Signaling, Retinoblastoma-binding Protein 9, Ulcerative Colitis

## Abstract

**Background & Aims:**

Retinoblastoma-binding protein 9 (RBBP9) was initially reported as cell cycle regulator via RB/E2F. Accumulating evidence has revealed the importance of RBBP9 in physiological and pathological states including inflammatory disease. However, the functional role of RBBP9 in ulcerative colitis (UC) and colitis-associated cancer (CAC) remains elusive.

**Methods:**

Human samples of UC and CAC were examined by immunohistochemical and bioinformatics analyses. We established dextran sodium sulfate (DSS)-induced colitis, azoxymethane (AOM)/DSS-induced CAC model, and *Apc*^*Min/+*^ sporadic tumor model using wild-type and *Rbbp9*^*-/-*^ mice. RNA sequencing was analyzed to identify the phenotype alternation upon *Rbbp9* deletion. In addition, genetic and pharmacological inhibition of the Janus kinase (JAK)/signal transducer and activator of transcription 1 (STAT1) pathway was performed.

**Results:**

The expression of RBBP9 was reduced in human UC and CAC samples. The loss of RBBP9 enhanced the activation of interferon (IFN)/JAK/STAT1 signaling, resulting in susceptibility to DSS-induced colitis and AOM/DSS-induced CAC tumors by increasing epithelial cell apoptosis and immune activation. An *in vitro* kinase assay revealed that RBBP9 directly regulated JAK/STAT1 signaling by suppressing STAT1 phosphorylation. A positive feedback loop involving epithelial cell apoptosis, commensal microbiome invasion, and activation of submucosal immune activity was identified in *Rbbp9*^*-/-*^ mouse intestines through enhanced JAK/STAT1 signaling in RBBP9-deficient epithelial cells and macrophages. The genetic inhibition of STAT1 or treatment with the JAK/STAT inhibitor reversed epithelial cell apoptosis and mitigated the enhanced susceptibility to DSS-induced colitis in *Rbbp9*^*-/-*^ mice.

**Conclusions:**

RBBP9 suppresses the intestinal inflammation by negatively regulating JAK/STAT1 signaling pathway.

## Graphical abstract

**Figure:**
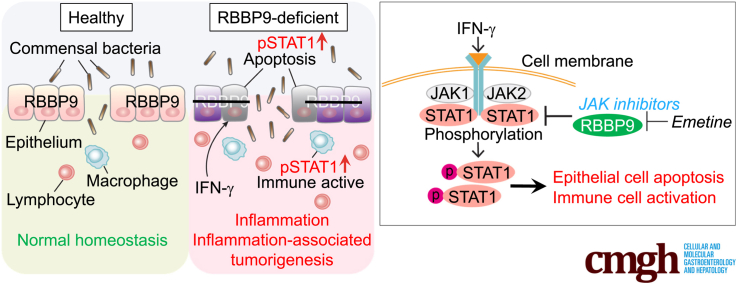

**Summary**

Loss of retinoblastoma-binding protein 9 (RBBP9) enhances Janus kinase/signal transducer and activator of transcription 1 signaling, increasing susceptibility to colitis and colitis-associated cancer. Reduced RBBP9 expression promotes epithelial cell apoptosis and immune activation. Signal transducer and activator of transcription 1 inhibition or upadacitinib treatment mitigates inflammation and tumor development in *Rbbp9*^*-/-*^ mice.

This article has an accompanying editorial.

Intestinal epithelial cells (IECs) form an epithelial layer that constitutes a central component of the mucosal barrier. Their dysfunction promotes microbial invasion from the gut lumen, which induces intestinal inflammation by activating the immune system in the lamina propria.^1^, ^2^, ^3^, ^4^, ^5^ Alterations in the normal function of IECs contribute to pathologies such as inflammatory bowel diseases (IBD), including Crohn’s disease and ulcerative colitis (UC), which are the main causes of colitis-associated cancer (CAC).^6^^,^^7^ Therefore, a deeper understanding of the regulatory systems controlling intestinal homeostasis and inflammation is essential. In this regard, pro-inflammatory cytokines, including tumor necrosis factor α (TNF-α) and interferons (IFNs). In addition to enhancing immune cell activities, these cytokines have been shown to contribute to epithelial barrier disruption in IBD by inducing IEC apoptosis^8^^,^^9^ and altering tight junction function.^10^ Although anti-TNF-α antibody therapy is one of the most established treatment options for patients with UC, some patients respond poorly to this treatment.^11^ On the other hand, although there have not been any approved anti-IFN treatments for UC, which is likely due to the undesirable effects of completely blocking the pleiotropic functions of IFNs, the Janus kinase (JAK)/signal transducer and activator of transcription (STAT) pathway inhibitors have been highlighted, especially for patients who do not respond well to anti-TNF-α therapy.^12^ Among the members of the STAT family, the expression and activation of STAT1 are predominantly upregulated in UC and may, therefore, play a critical role in the pathophysiology of colonic inflammation.^13^ A recent report has shown that the activation of the IFN-γ/JAK/STAT1 axis initiates a pro-apoptotic gene expression program and leads to epithelial stem cell death. Despite the well-established importance of the JAK/STAT1 pathway in inflammation, the regulatory mechanisms of this axis in IEC homeostasis and intestinal inflammation have not yet been fully elucidated.

Here, we address this biological problem in the context of the role and mechanism of action of retinoblastoma-binding protein 9 (RBBP9). RBBP9 is expressed in various human cancers.^14^, ^15^, ^16^, ^17^ Given its ability to bind to the RB protein, RBBP9 was initially reported to regulate RB/E2F cell cycle activity.^17^ More recent studies have identified RBBP9 as a serine hydrolase that promotes cancer progression by inhibiting the anti-proliferative function of transforming growth factor (TGF)-b in a pancreatic cancer model.^15^ Regarding inflammatory disorders, we recently determined a non-cell-autonomous function of RBBP9 as an interleukin (IL)-33-inducing damage-associated molecular pattern (DAMP) in the context of eosinophilic lung damage induced by *Strongyloides venezuelensis* infection.^18^ In this model, RBBP9 induced fibroblasts to produce PGE2, which led to the production of IL33. Thus, RBBP9-deficient mice showed reduced IL-33 induction following *Strongyloides venezuelensis* infection. Although accumulating evidence has revealed the importance of RBBP9 in physiological and pathological states, including inflammatory diseases, no previous studies have explored the role of RBBP9 in the development of intestinal inflammation and cancer.

In this study, through a series of *in vivo* and *ex vivo* experiments using RBBP9-knockout mice (*Rbbp9*^*-/-*^), along with bioinformatic and histological analyses of human UC and CRC samples, we have identified RBBP9 as a negative regulator of inflammation and inflammation-induced tumorigenesis in the intestine. Furthermore, we demonstrated the cell-autonomous activity of RBBP9 in suppressing the phosphorylation of STAT1. Consequently, genetic inhibition of STAT1 or the use of the JAK/STAT inhibitor upadacitinib (UPA) reversed the apoptotic phenotype of *Rbbp9*^*-/-*^ organoids and mitigated the enhanced colitis observed in dextran sodium sulfate (DSS)-treated *Rbbp9*^*-/-*^ mice. Thus, our findings shed light on the crucial role of RBBP9 in protecting against intestinal inflammation and inflammation-induced tumorigenesis by regulating the IFN/JAK/STAT1 signaling pathway.

## Results

### RBBP9 Expression is Downregulated in Samples From Patients With UC and CAC

**Figure 1:**
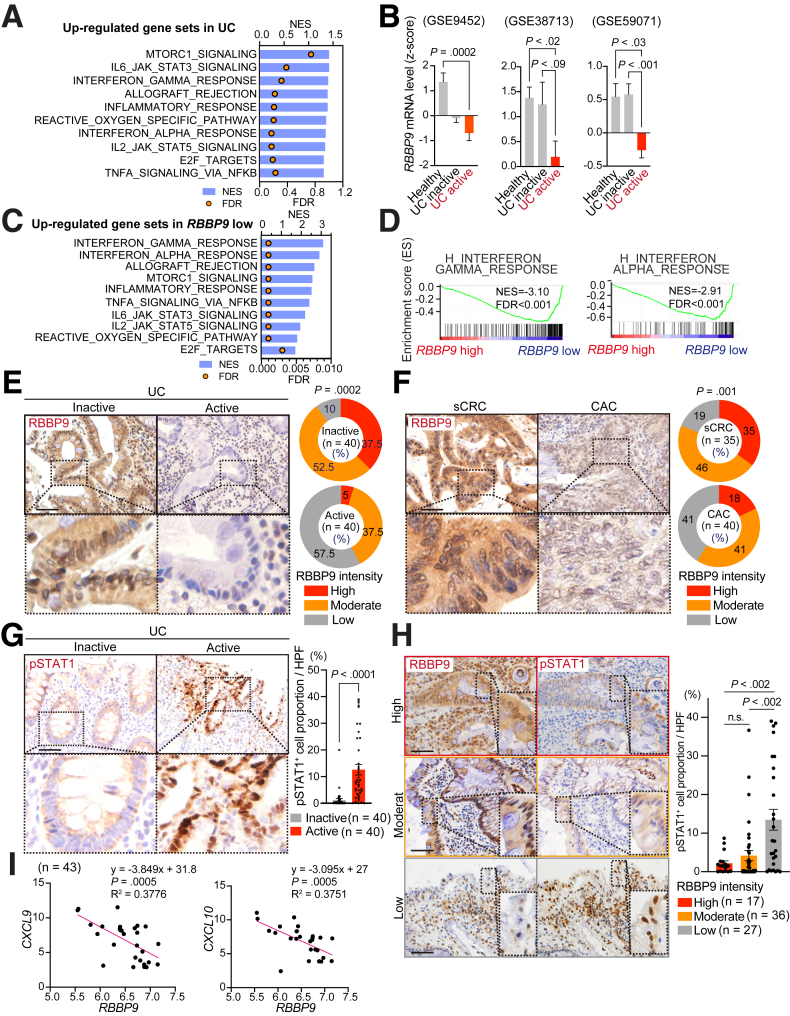
**Reduced expression of RBBP9 in patient samples of active UC and CAC was observed.** (*A*) GSEA of transcriptomic data for the indicated gene sets in active UC vs healthy patient samples in GSE38713 (n: healthy = 13, active UC = 15). (*B*) RNA expression levels of *RBBP9* in the indicated human samples from UC datasets (n: healthy = 5, inactive = 13, active = 8 in GSE9452; healthy = 13, inactive = 8, active = 15 in GSE38713; healthy = 11, inactive = 23, active = 74 in GSE59071). (*C, D*) GSEA of transcriptomic data (*C*) and enrichment plots (*D*) for the indicated gene sets in GSE38712, stratified based on correlation with *RBBP9* expression (n = 43). NES, normalized enrichment score; FDR, false-discovery rate. (*E*) Immunohistochemistry for RBBP9 (*left*) and quantification (*right*) in inactive or active UC lesions. (*F*) IHC for RBBP9 (*left*) and quantification (*right*) in sCRC or CAC. (*G*) IHC for pSTAT1 (*left*) and quantification (*right*) in inactive or active UC lesions. The positive cell proportion represents the percentage of pSTAT1-positive cells among all nuclei-positive cells. (*H*) IHC for RBBP9 or pSTAT1 (*left*), and quantification of pSTAT1-positive cells (*right*) in UC, stratified by RBBP9 intensity. (*I*) Correlation of *RBBP9* with the indicated genes in GSE38713. Scale bars, 50 μm. Mean ± SEM.

Transcriptomes from UC samples exhibited an increased expression of genes associated with inflammation and IFN signaling pathways compared with healthy tissues (Figure 1*A*), consistent with the critical role of the IFN pathway in the development of UC. In addition, UC samples showed enrichment of gene signatures related to IL6/JAK/STAT3 and mammalian target of rapamycin complex 1 (mTORC1) signaling, but not that of Wnt/b-catenin signaling (Figure 1*A*), suggesting the involvement of the IL6/JAK/STAT3 and mTORC1 signaling pathways in epithelial cell proliferation and regeneration in intestinal inflammation. Notably, further bioinformatics analyses revealed that active UC lesions showed lower *RBBP9* expression than healthy or inactive tissues from patients with UC during remission (Figure 1*B*). Correlation analyses demonstrated an association between low *RBBP9* expression and the enrichment of gene signatures associated with inflammation (“inflammatory_response” and “allograft_rejection”) and cell stress/damage response (“reactive_oxygen_species_pathway” and “apoptosis”) (Figure 1*C*). These data suggest a potential role of RBBP9 in controlling intestinal inflammation. In addition, IFN signaling-related signatures were enriched in the group with low *RBBP9* expression (Figure 1*D*). Furthermore, pathways associated with inflammation-mediated proliferation and regeneration, including “mTORC1_signaling” and “IL6/JAK/STAT3_signaling,” were negatively correlated with *RBBP9* expression (Figure 1*C*). These data suggest an inhibitory function of RBBP9 in the regulation of cell proliferation during inflammatory conditions.

In agreement with the results of the bioinformatics analyses, immunostaining of surgically resected samples from patients with UC revealed that RBBP9 expression was reduced in active inflammatory lesions compared to inactive healthy areas (Figure 1*E*). Sporadic colorectal cancer (sCRC) derived through the conventional adenoma-carcinoma sequence depends on the activation of the Wnt/β-catenin pathway. In contrast, inflammation-mediated proliferative pathways, including the mTORC and IL6/JAK/STAT3 signaling pathways, are more upregulated in CAC.^19^, ^20^, ^21^, ^22^, ^23^ Consistent with the above-described negative correlation between RBBP9 expression and the mTORC and STAT3 pathways in UC samples, RBBP9 expression in CAC samples was lower than that in sCRC samples (Figure 1*F*), suggesting different roles of RBBP9 in the development of these 2 types of CRC. Notably, active UC lesions exhibited a prominent increase in the proportion of phosphorylated STAT1 (pSTAT1)-positive cells compared with inactive areas (Figure 1*G*). Furthermore, we observed that the number of pSTAT1-positive cells was negatively correlated with RBBP9 intensity in UC samples (Figure 1*H*). Analyses of UC datasets revealed a negative correlation between the expression levels of *RBBP9* and IFN-related genes, including *CXCL9* and *CXCL10*, which are representative downstream targets of the IFN/JAK/STAT1 pathway (Figure 1*I*). Collectively, these results demonstrate a strong association between reduced RBBP9 expression, development of intestinal inflammation, and activation of the IFN signaling pathway.

### Loss of RBBP9 Renders the Intestines Susceptible to DSS-induced Colitis

**Figure 2:**
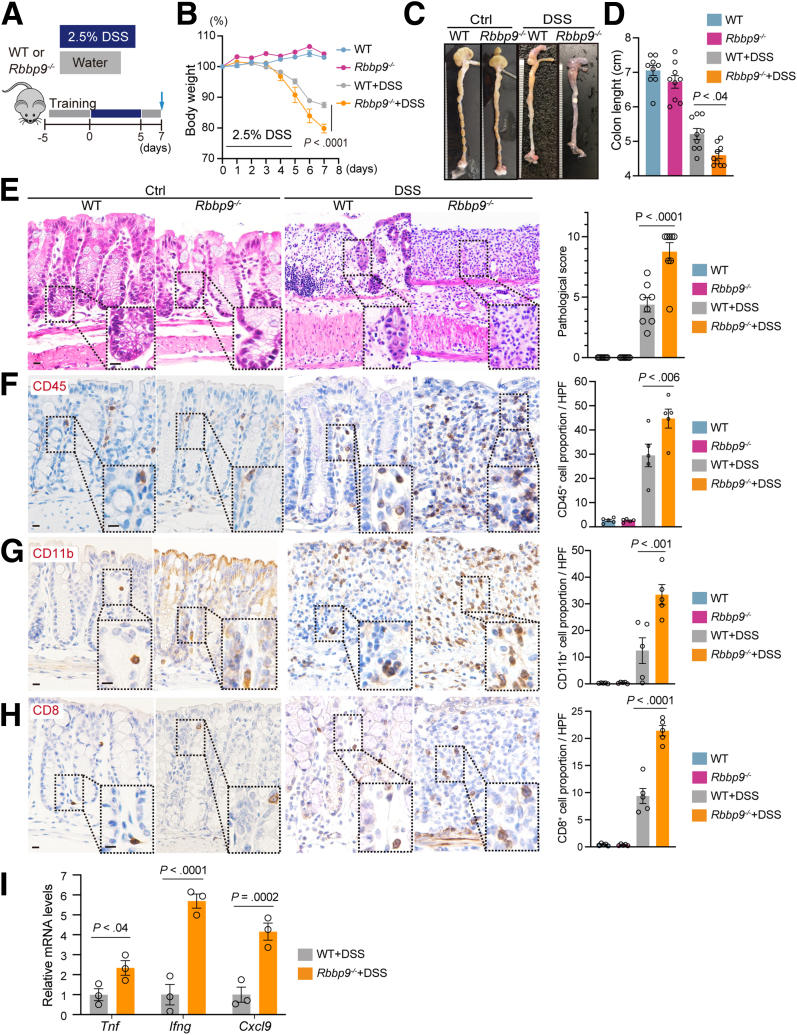
***Rbbp9***^***-/-***^**mice exhibit susceptibility to DSS-induced colitis.** (*A*) Schematic protocol of the DSS-induced colitis in WT and *Rbbp9*^*-/-*^ mice. (*B–E*) Percentage of change in body weight (*B*), representative images (*C*), and quantification (*D*) of colon length upon sacrifice, H&E staining and pathological scores (*E*) of WT and WT and *Rbbp9*^*-/-*^ colon sections (n = 8) in (*A*). Pathological score includes assessment of inflammation severity, crypt damage, and inflammatory extent. Scale bars, 50 μm. (*F–H*) Immunostaining and quantification of CD45 (*F*), CD11b (*G*), and CD8a (*H*) of WT and *Rbbp9*^*-/-*^ colon sections (n = 5) in (*A*). The positive cell proportion represents the percentage of immunostaining-positive cells among all nuclei-positive cells. Scale bars, 50 μm. (*I*) Relative mRNA expression levels of the indicated genes by qRT-PCR. Mean ± SEM.

To investigate whether RBBP9 deficiency sensitizes mice to colitis, we compared *Rbbp9*^*-/-*^ and control wild-type (WT) mice administered either normal water or DSS (Figure 2*A*). Although no differences were observed between genotypes without DSS treatment, *Rbbp9*^*-/-*^ mice exhibited significantly greater weight loss and colonic shortening than WT controls upon DSS treatment (Figure 2*B–D*), indicating the presence of severe inflammation in DSS-treated *Rbbp9*^*-/-*^ mice. Colonic sections from *Rbbp9*^*-/-*^ mice showed normal morphology compared with WT mice without DSS treatment; however, DSS-treated *Rbbp9*^*-/-*^ mice exhibited enhanced epithelial cell death, crypt destruction, extensive intestinal ulceration, and higher pathological scores compared with identically treated WT controls (Figure 2*E*). Furthermore, immunohistochemistry demonstrated increased infiltration of CD45^+^ leukocytes, CD11b^+^ myeloid cells, and CD8^+^ T cells in the colons of DSS-treated *Rbbp9*^*-/-*^ mice (Figure 2*F–H*). Consistent with these findings, quantitative real-time polymerase chain reaction (qRT-PCR) analyses revealed increased expression of inflammatory cytokine genes, including *Tnf* (TNF-α), *Ifng* (IFN-γ), and *Cxcl9* in *Rbbp9*^*-/-*^ colons (Figure 2*I*). These findings indicate that the loss of RBBP9 renders mice susceptible to experimentally induced colitis.

### Rbbp9^-/-^ mice exhibit enhanced Development of Colitis-associated Colon Tumors

**Figure 3:**
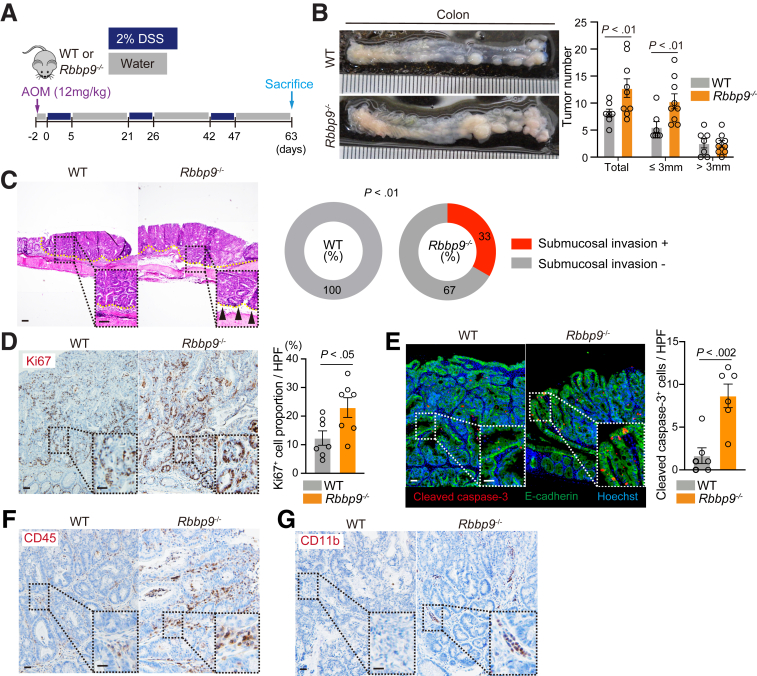
**Loss of RBBP9 enhances inflammation-induced tumorigenesis.** (*A*) Schematic protocol of the AOM/DSS-induced colorectal tumor model in WT and *Rbbp9*^*-/-*^ mice. (*B*) Total and size-dependent classified numbers of colon tumors in (A; n = 9). (*C*) Representative H&E staining (*left*) and proportion of submucosal invasion of tumors (*right*) of colonic sections in (*A*). *Yellow dashed lines* denote colon tumors. *Black arrowheads* denote invasive areas of the tumors. Scale bars, 100 μm. (*D*) Immunostaining of Ki67 and its quantification in AOM/DSS-induced tumors (n = 7). The positive cell proportion represents the percentage of immunostaining-positive cells among all nuclei-positive cells. Scale bars, 50 μm. (*E*) Co-immunostaining of cleaved caspase-3 and E-cadherin and quantification in AOM/DSS-induced tumors (n = 7). Scale bars, 50 μm. (*F, G*) Immunostaining of CD45 (*F*) and CD11b (*G*). Scale bars, 50 μm.

As inflammation is a widely recognized promoter of intestinal tumorigenesis, we examined whether the loss of RBBP9 could contribute to intestinal cancer in mice. To test this hypothesis, we employed a well-established CAC model that combines the carcinogen azoxymethane (AOM) and DSS (Figure 3*A*). Notably, AOM/DSS-treated *Rbbp9*^*-/-*^ mice developed more colonic tumors accompanied by an invasive carcinoma area than identically treated control mice (Figure 3*B* and *C*). Moreover, tumors that developed in *Rbbp9*^*-/-*^ mice exhibited higher rates of epithelial cell proliferation and death than those in the control mice (Figures 3*D* and *E*). Consistent with enhanced inflammation in DSS-treated *Rbbp9*^*-/-*^ mice (Figure 2), AOM/DSS-treated *Rbbp9*^*-/-*^ tumors exhibited increased infiltration of immune cells, including CD45^+^ leukocytes and CD11b^+^ myeloid cells (Figure 3*F* and *G*), than the controls. We also observed a significant increase in the number of small tumors (≤3 mm in diameter) in *Rbbp9*^*-/-*^ mice, whereas the number of large tumors (>3 mm) was not significantly altered (Figure 3*B*), suggesting that the loss of RBBP9 enhances the initiation of inflammation-induced tumorigenesis rather than tumor growth.

### Loss of RBBP9 Promotes Inflammation-mediated Proliferation

**Figure 4:**
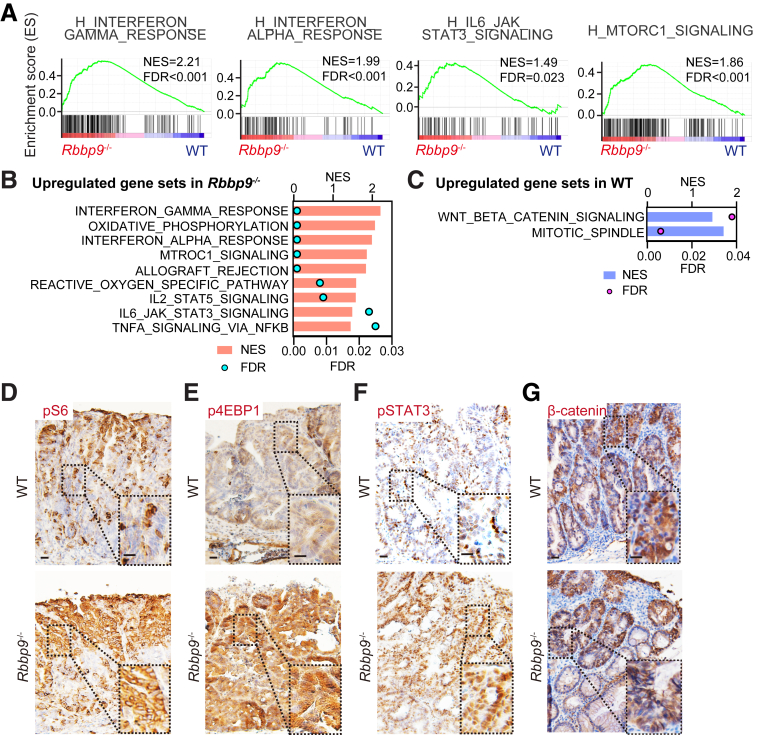
**Inflammation-mediated proliferation pathways are upregulated in tumors of AOM/DSS-treated *Rbbp9***^***-/-***^**mice.** (*A–C*) GSEA of RNA-seq data from AOM/DSS-induced tumors in WT and *Rbbp9*^*-/-*^ colons (n = 3 for each group). (*A*) GSEA plots in *Rbbp9*^*-/-*^ vs WT colon tumor. FDR, False discovery rate; NES, normalized enrichment score. Upregulated gene sets in *Rbbp9*^*-/-*^ (*B*) and in WT tumors (*C*). FDR, False-discovery rate. (*D–G*) Representative immunostaining of pS6 (*D*), p4EBP1 (*E*), pSTAT1 (*F*), and β-catenin (*G*) in AOM/DSS-induced tumors (n = 3). Scale bars, 50 μm. Mean ± SEM.

To determine the mechanism underlying the pro-tumor phenotype of *Rbbp9*^*-/-*^ mice in the CAC model, we conducted an unbiased transcriptomic analysis (RNA sequencing [RNA-seq]) of tumors from AOM/DSS-treated *Rbbp9*^*-/-*^ mice. *Rbbp9*^*-/-*^ tumors revealed the positive enrichment of pathways related to inflammation and cellular stress (“TNF-α_signaling_via_NFκB,” “allograft_rejection,” “oxidative_phosphorylation,” and “reactive_oxygen_specific_pathway”) and IFN signaling (“interferon_alpha_response” and “interferon_gamma_response”) (Figure 4*A* and *B*). Additionally, gene signatures associated with inflammation-mediated proliferation, such as the “mTORC1_signaling” and “IL6_JAK_STAT3_signaling” pathways, were upregulated in AOM/DSS-treated *Rbbp9*^*-/-*^ tumors (Figure 4*A* and *B*).^24^ On the other hand, signatures related to “mitotic_spindle” and “Wnt_β-catenin_signaling,” which are critical pathways in the development of sporadic CRC, were even downregulated in AOM/DSS-treated *Rbbp9*^*-/-*^ tumors (Figure 4*C*). Consistent with these results, immunohistochemistry for phosphorylated S6 (pS6) and phosphorylated 4EBP1 (p4EBP1), which are downstream molecules of the mTORC1 pathway, as well as pSTAT3, revealed activation of both mTORC1 and IL6/JAK/STAT3 pathways in AOM/DSS-treated *Rbbp9*^*-/-*^ tumors (Figure 4*D–F*). In contrast, nuclear β-catenin staining was not increased in these tumors (Figure 4*G*). These data suggest that the enhanced tumor phenotypes in RBBP9-deficient mice do not depend on Wnt/β-catenin signaling activation but rather on the mTORC1 and IL6/JAK/STAT3 signaling pathways. This is consistent with previous reports indicating that mutations in Wnt/β-catenin pathway-related genes and the consequent activation of this pathway are less frequent in CAC than in sporadic CRC.^20^ Instead, the mTORC1 and IL6/JAK/STAT3 signaling pathways appear to be predominant in CAC development.

### Enhanced Inflammation Upon RBBP9 Loss Exacerbates Reduced Tumor Development in Apc^Min/+^ Mice

**Figure 5:**
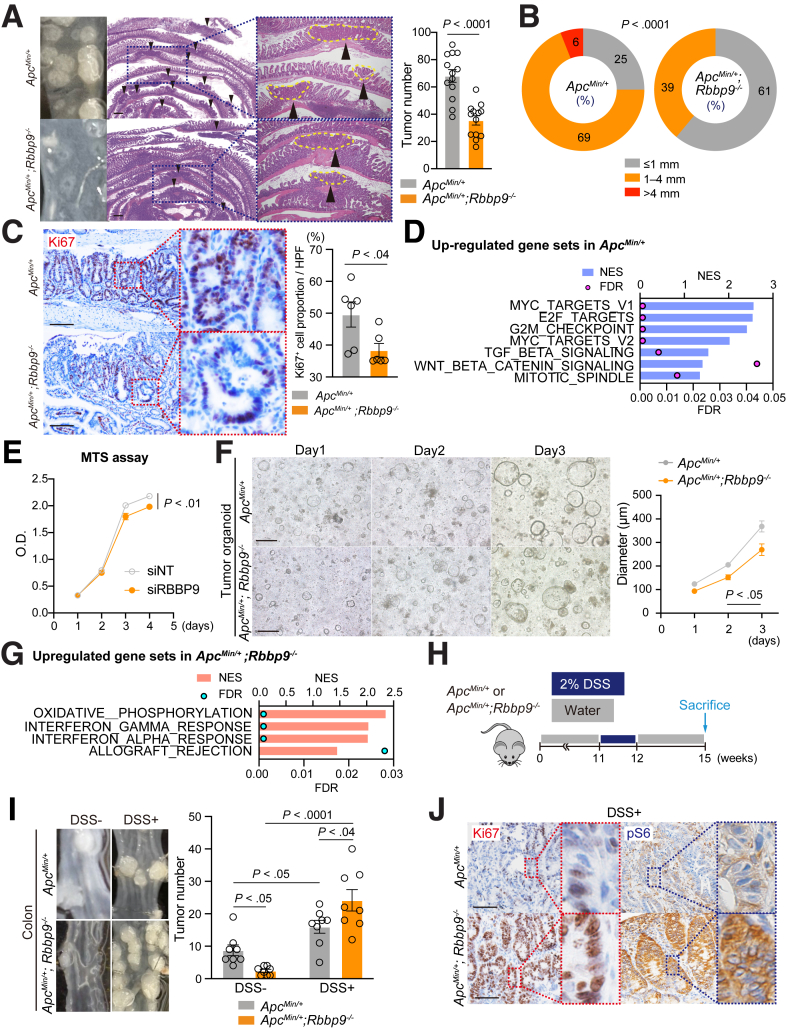
**Enhanced inflammation due to loss of RBBP9 overrides the reduced tumor development in the *Apc***^***Min/+***^**mouse model.** (*A, B*) Macroscopic pictures, H&E staining, and total numbers of small intestinal tumors (*A*), and tumor size distribution (*B*) in *Apc*^*Min/+*^ and *Apc*^*Min/+*^*; Rbbp9*^*-/-*^ mice (n = 14). Scale bars, 500 μm. (*C*) Immunostaining and quantification of Ki67 in *Apc*^*Min/+*^ and *Apc*^*Min/+*^*; Rbbp9*^*-/-*^ mouse tumors (n = 6). The positive cell proportion represents the percentage of immunostaining-positive cells among all nuclei-positive cells. Scale bars, 100 μm. (*D*) GSEA of RNA-seq data from *Apc*^*Min/+*^ and *Apc*^*Min/+*^*; Rbbp9*^*-/-*^ mouse tumors (n = 3). Upregulated gene sets in *Apc*^*Min/+*^. FDR, False-discovery rate. (*E*) MTS assay measuring the growth of MC38 cells treated with siNT or siRBBP9 over the indicated days (n = 5 for each group). (*F*) Representative images of tumor organoids derived from *Apc*^*Min/+*^ and *Apc*^*Min/+*^*; Rbbp9*^*-/-*^ small intestines on days 1, 2, and 3. (*left*, n = 9 for each group) and diameter of organoids in (*right*; n = 9 for each group). Scale bars, 200 μm. (*G*) Upregulated gene sets in *Apc*^*Min/+*^*;Rbbp9*^*-/-*^ tumors. FDR, False-discovery rate. (*H*) Schematic protocol of the DSS-induced colitis experiment in *Apc*^*Min/+*^ and *Apc*^*Min/+*^*; Rbbp9*^*-/-*^ mice. (*I, J*) Total numbers (*I*; n = 8) and immunostaining for the specified antibodies (*J*) of colorectal tumors in *Apc*^*Min/+*^ and *Apc*^*Min/+*^*; Rbbp9*^*-/-*^ treated with or without DSS. Scale bars, 50 μm. Mean ± SEM.

Next, we investigated the effect of RBBP9 loss on intestinal tumorigenesis without overt inflammation. We crossed *Rbbp9*^*-/-*^ mice with *Apc*^*Min/+*^ mice, in which tumors spontaneously develop in the small intestine and colon without an exogenous inflammatory stimulus. Previous reports in other cancer models have demonstrated the pro-tumor function of RBBP9 through either the inhibition of the cell cycle regulatory protein RB or the serine hydrolase activity-mediated suppression of the TGF-β pathway.^15^^,^^17^ In agreement with these reports, *Apc*^*Min/+*^;*Rbbp9*^*-/-*^ mice developed fewer and smaller tumors in the small intestine, which is the dominant location of tumor occurrence in the *Apc*^*Min/+*^ mouse model, with reduced proliferative activity compared with control mice (Figure 5*A–C*). RNA-seq data of spontaneously developed tumors in *Apc*^*Min/+*^ mice and *Apc*^*Min/+*^;*Rbbp9*^*-/-*^ mice demonstrated that cell cycle-related gene signatures, such as the “Myc_targets,” “E2F_targets,” “G2M_checkpoint,” “and "mitotic_spindle" pathways, were upregulated in tumors of *Apc*^*Min/+*^ mice (Figure 5*D*). Consistent with these results, we observed reduced growth of both the RBBP9-suppressed colon cancer cell line (MC38) and tumor organoids generated from *Apc*^*Min/+*^;*Rbbp9*^*-/-*^ small intestinal tumors compared with their respective controls with normal RBBP9 expression (Figure 5*E* and *F*). Interestingly, *Apc*^*Min/+*^;*Rbbp9*^*-/-*^ tumors exhibited the enrichment of gene signatures associated with inflammation and IFN signaling, including “oxidative_phosphorylation,” “allograft_rejection,” “interferon_alpha_response,” and “interferon_gamma_response” (Figure 5*G*), but not inflammation-mediated proliferative signatures. These data suggest that pro-inflammatory changes in *Apc*^*Min/+*^;*Rbbp9*^*-/-*^ tumors are only evident at the transcriptomic level and may not be sufficient to generate proliferative activity in the absence of inflammatory stimuli. To test this hypothesis, we treated *Apc*^*Min/+*^;*Rbbp9*^*-/-*^ mice with DSS and compared the number of colon tumors between *Apc*^*Min/+*^ and *Apc*^*Min/+*^;*Rbbp9*^*-/-*^ mice, as DSS-induced inflammation mainly affects the colon (Figure 5*H*). Consistent with the small intestinal tumors, the number of colon tumors was lower in *Apc*^*Min/+*^;*Rbbp9*^*-/-*^ mice than in control mice (Figure 5*I*). Consistent with a previous report demonstrating that the addition of inflammatory stimuli enhances tumor development in an *Apc*^*Min/+*^ mouse model,^25^ the number of colon tumors increased in both *Apc*^*Min/+*^ and *Apc*^*Min/+*^;*Rbbp9*^*-/-*^ mice (Figure 5*I*). Importantly, DSS-treated *Apc*^*Min/+*^;*Rbbp9*^*-/-*^ mice exhibited a significantly higher number of colon tumors than identically treated *Apc*^*Min/+*^ mice, accompanied by increased Ki67 and pS6 staining, indicating stronger inflammation-mediated proliferative activity (Figure 5*I* and *J*). These data suggest that the loss of RBBP9 enhances inflammation and inflammation-mediated cell proliferation in response to inflammatory stimuli, thereby overcoming the reduction in spontaneous tumorigenesis observed in *Apc*^*Min/+*^;*Rbbp9*^*-/-*^ mice.

### RBBP9 Loss Augments the IFN-γ-induced JAK/STAT1 Pathway, Enhancing Epithelial Cell Apoptosis

**Figure 6:**
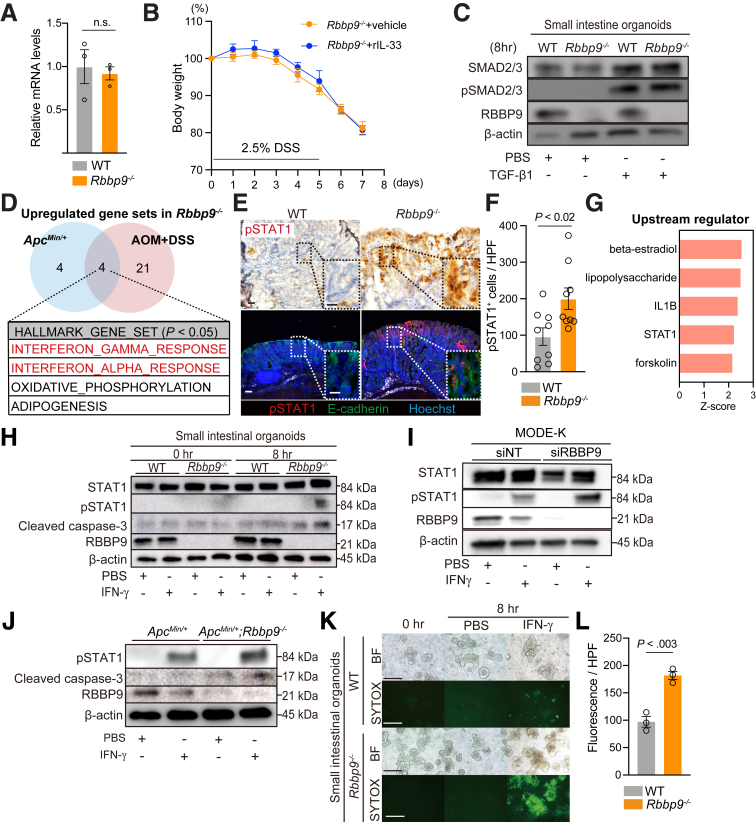
**Loss of RBBP9 enhanced IFN-γ-induced apoptosis in intestinal epithelium.** (*A*) Relative mRNA levels of *Il33* analyzed using qRT-PCR (n = 3 for each group). n.s., Not significant. (*B*) Percentage of change in body weight of *Rbbp9*^*-/-*^ mice treated either with vehicle or recombinant IL33 (n = 6 for each group). (*C*) Immunoblotting for the indicated proteins in WT and *Rbbp9*^*-/-*^ intestinal organoids treated with either PBS or TGF-β1 (10 ng/mL) for 8 hours. (*D*) Common gene sets upregulated in *Apc*^*Min/+*^*; Rbbp9*^*-/-*^ tumors (vs *Apc*^*Min/+*^ tumors); and in AOM/DSS-treated *Rbbp9*^*-/-*^ tumors (vs WT tumors) among the HALLMARK sets in GSEA. (*E, F*) Immunostaining (*E, top*) and quantification (*F*) of pSTAT1 and co-immunostaining of pSTAT1 and E-cadherin (*E, bottom*) in AOM/DSS-induced tumors in WT and *Rbbp9*^*-/-*^ mice. Scale bars, 50 μm (*E, top*), 100 μm (*E, bottom*). (*G*) The “Upstream Regulator” analysis in Ingenuity Pathway Analysis (IPA) on RNA-seq data of AOM/DSS-induced tumors. The absolute z-score, representing the degree of activation, is expressed among the total genes assigned to the activated upstream regulator by IPA. (*H*) Immunoblotting for the indicated proteins in WT and *Rbbp9*^*-/-*^ intestinal organoids treated with PBS or IFN-γ (10 ng/mL). (*I*) Immunoblotting for the indicated proteins in siNT- or siRBBP9-treated MODE-K cells stimulated with PBS or IFN-γ (10 ng/mL) for 4 hours. (*J*) Immunoblotting for the indicated proteins in *Apc*^*Min/+*^ and *Apc*^*Min/+*^*; Rbbp9*^*-/-*^ tumor organoids stimulated with PBS or IFN-γ (10 ng/mL) for 8 hours. (*K, L*) Representative images of bright field microscopy (BF) and SYTOX Green staining (*K*) and quantification (*L*) of WT and *Rbbp9*^*-/-*^ organoids treated with PBS or IFN-γ (10 ng/mL) (n = 3). Scale bars, 200 μm.

Our previous work identified the non-cell-autonomous function of RBBP9 as a DAMP that activates fibroblasts to produce IL33. IL33-deficient mice are susceptible to DSS-induced colitis,^26^ similar to *Rbbp9*^*-/-*^ mice. Therefore, to determine the mechanism by which RBBP9 suppresses the pro-inflammatory state in the intestine, we first examined whether the expression levels of IL33 were changed in *Rbbp9*^*-/-*^ mice. We did not observe a significant change in IL33 expression between genotypes (Figure 6*A*). Furthermore, the addition of recombinant IL33 did not cause any change in DSS-induced colitis in *Rbbp9*^*-/-*^ mice (Figure 6*B*). The serine hydrolase activity of RBBP9 was reported to suppress the TGF-β signaling pathway in pancreatic cancer cell lines.^15^ Because the activation of the TGF-β/Suppressor of Mothers against Decapentaplegic (SMAD) axis mediates epithelial cell apoptosis,^27^^,^^28^ we investigated the phosphorylation status of SMAD2/3 (pSMAD2/3) in RBBP9-deficient epithelial organoids generated from the small intestines of *Rbbp9*^*-/-*^ mice. The levels of pSMAD2/3 were comparable between WT and *Rbbp9*^*-/-*^ organoids under basal and TGF-β-stimulated conditions (Figure 6*C*). These data from the analyses using genetically knockout mice for RBBP9 suggest that RBBP9 plays a distinct role in suppressing intestinal inflammation.

To determine the signaling pathways through which RBBP9 loss enhances intestinal inflammation and inflammation-mediated proliferation, we focused on the overlapping gene signatures between the RNA-seq data from the AOM/DSS and *Apc*^*Min/+*^ tumor models (Figure 6*D*). We identified 4 overlapping signatures that were significantly upregulated in *Rbbp9*^*-/-*^ mice: “interferon_gamma_response,” “interferon_alpha_response,” “oxidative_phosphorylation,” and “adipogenesis.” In addition, we observed a clear increase in pSTAT1 expression in AOM/DSS-induced tumors in *Rbbp9*^*-/-*^ mice (Figure 6*E* and *F*). The “Upstream Regulator” analysis in the Ingenuity Pathway Analysis predicted STAT1 as a potential activated upstream regulator in *Rbbp9*^*-/-*^ samples (Figure 6*G*). To test whether RBBP9 loss augments the inflammatory response by activating STAT1 phosphorylation, we stimulated WT and *Rbbp9*^*-/-*^ organoids with IFN-γ. Notably, we observed an increase in pSTAT1 levels in *Rbbp9*^*-/-*^ organoids treated with IFN-γ, whereas the levels of total STAT1 remained unchanged (Figure 6*H*). Similarly, both RBBP9-suppressed IECs (MODE-K) and *Apc*^*Min/+*^;*Rbbp9*^*-/-*^ tumor organoids showed increased pSTAT1 levels when treated with IFN-γ compared with the respective controls with normal RBBP9 expression (Figure 6*I* and *J*). In agreement with previous reports showing that the activation of the IFN/JAK/STAT1 axis induces cell apoptosis in the intestinal epithelium and organoids,^8^^,^^9^ the level of cleaved caspase-3 was increased in *Rbbp9*^*-/-*^ organoids and *Apc*^*Min/+*^;*Rbbp9*^*-/-*^ tumor organoids treated with IFN-γ, concomitant with increased pSTAT1 levels (Figure 6*H* and *J*). Consistent with these data, we found that *Rbbp9*^*-/-*^ organoids exhibited a disrupted and dark appearance after IFN-γ treatment (Figure 6*K*). The proportion of dying organoids positive for SYTOX Green was higher in *Rbbp9*^*-/-*^ organoids than the controls upon IFN-γ treatment (Figure 6*K* and *L*), indicating that increased activity of the JAK/STAT1 axis augments cell death in *Rbbp9*^*-/-*^ organoids.

### RBBP9 Loss Impacts Epithelial Barrier Function, Further Exacerbating Intestinal Inflammation

**Figure 7:**
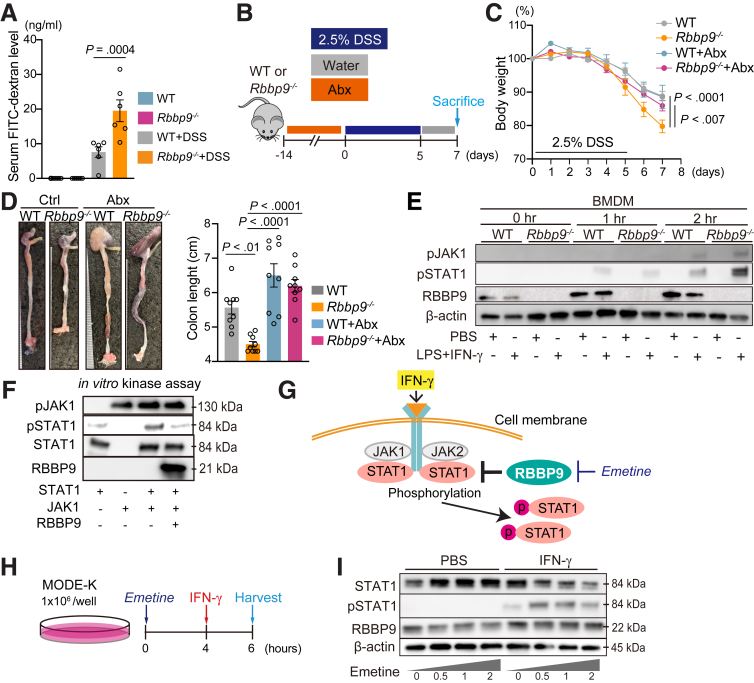
**RBBP9 interferes with the IFN-γ-induced JAK/STAT1 pathway by inhibiting STAT1 phosphorylation.** (*A*) Quantitative serum FITC-dextran in DSS-treated WT and *Rbbp9*^*-/-*^ mice. (*B*) Schematic protocol of the DSS-induced colitis pretreated with antibiotic (Abx) cocktails in WT and *Rbbp9*^*-/-*^ mice. (*C, D*) Percentage of change in body weight (*C*), images and quantification (*D*) of colon length (n = 9) upon sacrifice in (*B*). (*E*) Immunoblotting for the indicated proteins in BMDMs from WT and *Rbbp9*^*-/-*^ mice, stimulated with PBS or IFN-γ (10 ng/mL) for 2 hours. (*F*) In vitro kinase assay of recombinant STAT1 by recombinant JAK1 with or without the presence of recombinant RBBP9, followed by immunoblotting for the indicated proteins. (*G*) Schematics illustrating that RBBP9 regulates the IFN/JAK/STAT1 pathway by inhibiting STAT1 phosphorylation. (*H, I*) Schematics depicting MODE-K cells stimulated with either PBS or IFN-γ (10 ng/mL) for 4 hours, with or without the presence of emetine (*H*). Immunoblotting for the indicated proteins (*I*). Mean ± SEM.

The loss of epithelial barrier function leads to the invasion of the gut microbiota into the lamina propria, where microbes encounter phagocytes such as macrophages. To directly examine whether the loss of RBBP9 impacts epithelial barrier function, we quantified the permeability by orally administering fluorescein isothiocyanate (FITC)-dextran to WT and *Rbbp9*^*-/-*^ mice and measuring the serum levels. Notably, although there was no change in serum FITC levels between genotypes without DSS treatment, DSS-treated *Rbbp9*^*-/-*^ mice showed higher FITC levels than WT mice, indicating that RBBP9 loss exacerbates intestinal permeability by further impairing epithelial barrier function upon DSS treatment (Figure 7*A*). Furthermore, consistent with the notion that the enhanced susceptibility of *Rbbp9*^*-/-*^ mice to DSS-induced colitis is attributed to increased bacterial invasion due to impaired barrier function, treatment with broad-spectrum antibiotics abolished the enhanced body weight loss and shortened colon length observed in DSS-treated *Rbbp9*^*-/-*^ mice (Figure 7*B–D*). To examine the effect of RBBP9 loss on macrophages, we collected bone marrow-derived macrophages (BMDMs) and stimulated them with lipopolysaccharide (LPS), a component of the bacterial wall.^29^ This treatment strongly induced pSTAT1 expression in *Rbbp9*^*-/-*^ BMDMs upon stimulation with IFN-γ (Figure 7*E*). To further understand the underlying mechanisms by which RBBP9 regulates the levels of STAT1 phosphorylation, we performed *in vitro* kinase assays using recombinant JAK1 and STAT1 in the presence or absence of RBBP9. Notably, the JAK1-mediated increase in pSTAT1 levels reverted to autophosphorylation upon the addition of recombinant RBBP9 (Figure 7*F*), suggesting the direct inhibitory effect of RBBP9 on STAT1 phosphorylation (Figure 7*G*). The bioactive natural product emetine has been reported as a selective inhibitor of the enzymatic activity of RBBP9.^14^^,^^30^ We found that treatment with emetine increased the IFN-induced phosphorylation of STAT1 in MODE-K cells (Figure 7*H* and *I*), similar to the results observed in RBBP9-suppressed MODE-K cells (Figure 6*I*). Collectively, these results support a model in which the loss of RBBP9 results in JAK/STAT1-mediated apoptosis of epithelial cells, contributing to epithelial barrier dysfunction, which, in turn, promotes commensal bacterial infiltration and exacerbates inflammation.

### STAT1 Inhibition Reverses the Increased Susceptibility of Rbbp9^-/-^ Mice to Colitis

**Figure 8:**
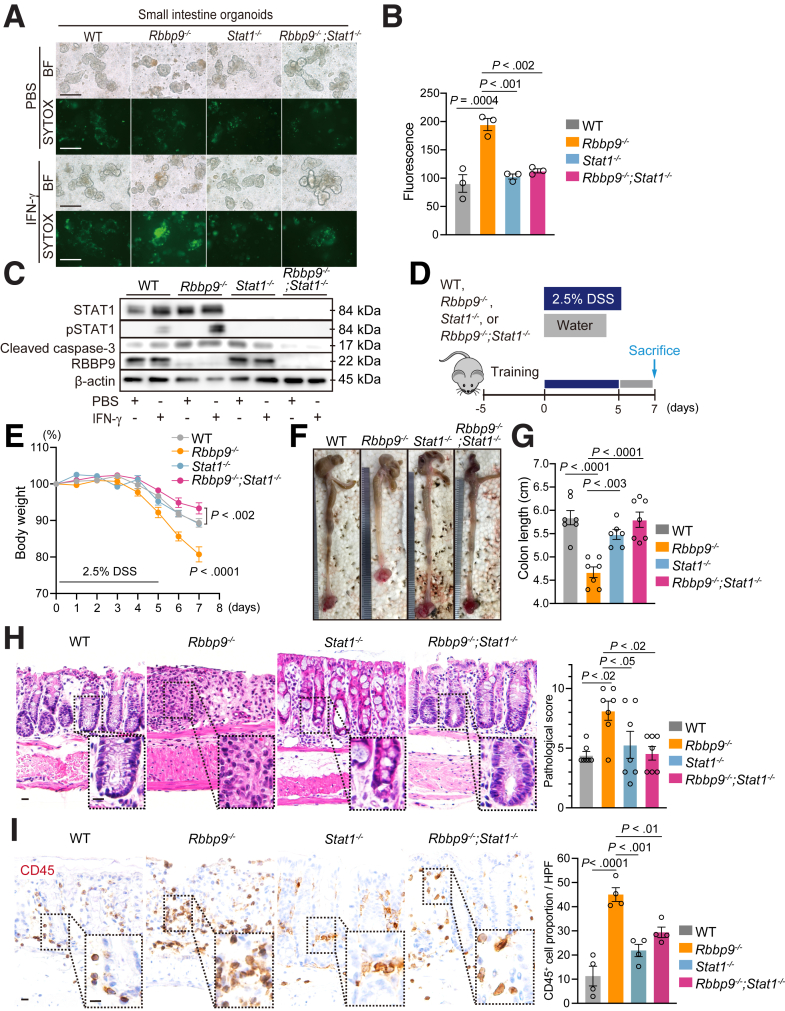
**Simultaneous loss of STAT1 alleviates epithelial cell apoptosis and mitigates colitis exacerbation in *RBBP9***^***-/-***^**mice.** (*A, B*) Representative images (*A*) of bright field microscopy (BF) and SYTOX green staining and quantification (*B*) of intestinal organoids derived from mice with the indicated genotypes, treated with either PBS or IFN-γ (10 ng/mL) for 8 hours (n = 3). Scale bars, 200 μm. (*C*) Immunoblotting for the indicated proteins in intestinal organoids derived from WT, *Rbbp9*^*-/-*^, *Stat1*^*-/-*^, and *Rbbp9*^*-/-*^*;Stat1*^*-/-*^ mice, treated with PBS or IFN-γ (10 ng/mL) for 8 hours. (*D*) Schematic protocol of DSS-induced colitis. (*E–I*) Percentage of change in body weight (*E*), representative images (*F*), and quantification (*G*) of colon length upon sacrifice, H&E staining and pathological scores (*H*) of mouse colon sections from the indicated genotypes (n = 6 for each group) in (*D*). Pathological score includes assessment of inflammation severity, crypt damage, and inflammatory extent. Scale bars, 50 μm. Mean ± SEM. (*I*) Immunostaining of CD45 in colon sections from mice with the indicated genotypes subjected to a DSS-induced colitis model, and quantification (n = 4 for each group). Scale bars, 50 μm.

Given the critical role of RBBP9 in directly suppressing the phosphorylation of STAT1, we examined whether the loss of STAT1 reversed the apoptotic phenotype of epithelial cells and rescued the increased susceptibility of *Rbbp9*^*-/-*^ mice to experimental colitis. To achieve this, we crossed *Rbbp9*^*-/-*^ mice with STAT1-knockout (*Stat1*^*-/-*^) mice to generate double-knockout (*Rbbp9*^*-/-*^*;Stat1*^*-/-*^) mice. There was no difference in the survival of the organoids generated from any of the genotypes under basal conditions (Figure 8*A*). Notably, the increased proportion of dying organoids positive for SYTOX Green in the *Rbbp9*^*-/-*^ group reverted to WT levels in *Rbbp9*^*-/-*^*;Stat1*^*-/-*^ organoids upon IFN-γ treatment (Figure 8*A* and *B*), indicating that inhibiting the JAK/STAT1 axis alleviated the augmented cell death in *Rbbp9*^*-/-*^ organoids. Immunoblotting results also demonstrated that *Rbbp9*^*-/-*^*;Stat1*^*-/-*^ organoids showed reduced levels of cleaved caspase-3 compared with *Rbbp9*^*-/-*^ organoids when treated with IFN-γ, concomitant with the abolished phosphorylation of STAT1 (Figure 8*C*). To examine whether the enhanced inflammatory phenotypes in *Rbbp9*^*-/-*^ mice were reversed by the simultaneous loss of STAT1, *Rbbp9*^*-/-*^*;Stat1*^*-/-*^ mice were treated with DSS (Figure 8*D*). Notably, the enhanced loss of body weight and shortened colon length observed in DSS-treated *Rbbp9*^*-/-*^ mice reverted to levels similar to WT controls in *Rbbp9*^*-/-*^*;Stat1*^*-/-*^ mice (Figure 8*E–G*). Furthermore, the increased pathological severity of colitis and CD45^+^ leukocyte infiltration in DSS-treated *Rbbp9*^*-/-*^ mice was completely abolished by the simultaneous loss of STAT1 in *Rbbp9*^*-/-*^*;Stat1*^*-/-*^ mice (Figure 8*H* and *I*). These data indicated that inhibition of the JAK/STAT1 axis reversed epithelial cell apoptosis, alleviating the pro-inflammatory state in *Rbbp9*^*-/-*^ mouse intestines.

### The JAK1 Inhibitor UPA Alleviated the Enhanced Colitis in Rbbp9^-/-^ Mice

**Figure 9:**
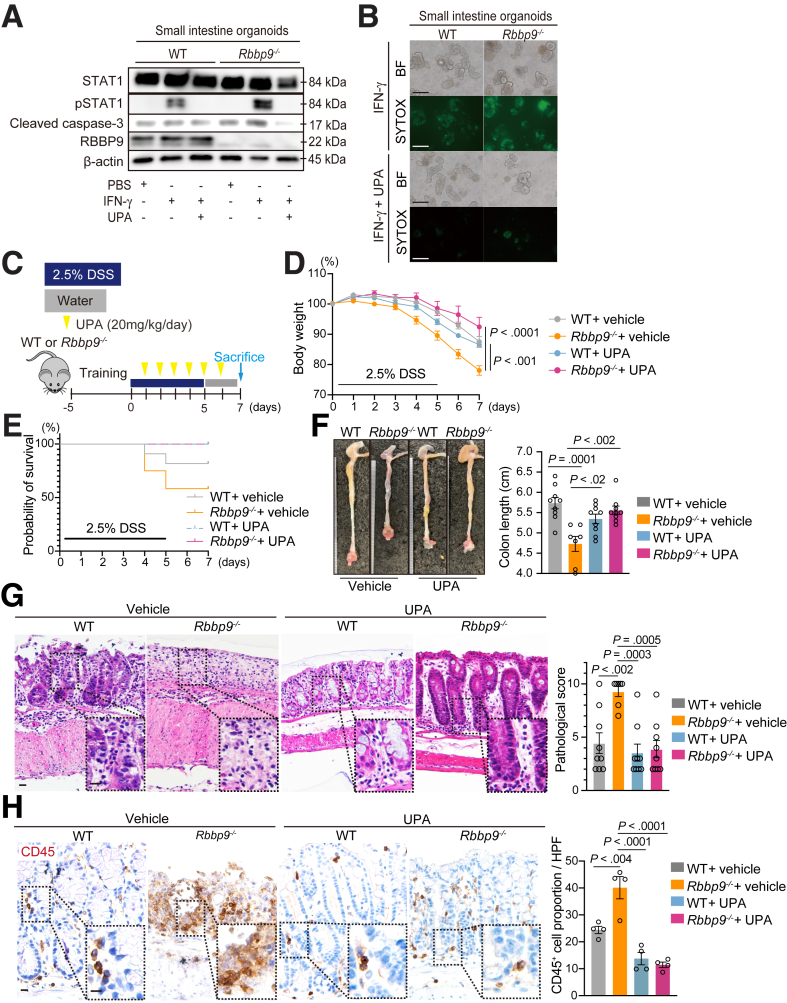
**Upadacitinib ameliorates enhanced inflammation in *RBBP9***^***-/-***^**mice in the DSS colitis model.** (*A*) Immunoblotting for the indicated antibodies in WT and *Rbbp9*^*-/-*^ intestinal organoids stimulated with PBS or IFN-γ (10 ng/mL) in the presence or absence of UPA (50 nM) for 8 hours. (*B*) Representative images of bright field microscopy (BF) and SYTOX green staining of WT and *Rbbp9*^*-/-*^ intestinal organoids treated with IFN-γ (10 ng/mL) in the presence or absence of UPA (50 nM) for 8 hours. Scale bars, 200 μm. (*C*) Schematic protocol of DSS-induced colitis with UPA treatment in WT and *Rbbp9*^*-/-*^ mice. (*D–H*) Percentage of change in body weight (*D*), Kaplan-Meier survival curve (*E*), images and quantification (*F)* of colon length upon sacrifice, H&E staining and pathological scores (*G*) of colon sections from the indicated genotypes (n = 8) in (*C*). Pathological score includes assessment of inflammation severity, crypt damage, and inflammatory extent. Scale bars, 50 μm. (*H*) Immunostaining of CD45 in colon sections from WT and *Rbbp9*^*-/-*^ mice treated with either vehicle or UPA (20 mg/kg/day) in the DSS-induced colitis model. Scale bars, 50 μm.

JAK inhibitors can potentially affect multiple cytokine-dependent pathways involved in UC pathology, which could theoretically increase the risk of toxicity and adverse events. Therefore, given the chronic nature of UC, there is a need for *in vivo* target specificity and an understanding of the long-term safety consequences of treatment. Key molecular and cellular patterns driving individual diseases must also be identified to predict the best therapeutic approach for patients with UC. UPA, a recently approved JAK inhibitor with a high specificity for JAK1, has shown efficacy in inducing and maintaining remission in patients with UC.^12^ Given the function of RBBP9 in regulating STAT1 phosphorylation, which is mainly targeted by JAK1, we used UPA to examine whether it effectively reduced the increased apoptotic phenotype observed in *Rbbp9*^*-/-*^ organoids. Treatment with UPA suppressed cleaved caspase-3 levels upon IFN-γ treatment in *Rbbp9*^*-/-*^ organoids (Figure 9*A*). Furthermore, the number of SYTOX-positive dying *Rbbp9*^*-/-*^ organoids was reduced upon treatment with UPA (Figure 9*B*).

Finally, we examined whether UPA treatment effectively attenuated the susceptibility of *Rbbp9*^*-/-*^ mice to DSS-induced colitis (Figure 9*C*). Notably, the increased body weight loss and impaired survival observed in DSS-treated *Rbbp9*^*-/-*^ mice reverted to the levels observed in WT controls upon UPA treatment (Figure 9*D* and *E*). Furthermore, the shortened colon length of DSS-treated *Rbbp9*^*-/-*^ mice reverted to levels similar to the WT control, concomitant with the amelioration of histological colitis in DSS-treated *Rbbp9*^*-/-*^ mice upon UPA treatment (Figure 9*F* and *G*). Additionally, CD45^+^ leukocyte infiltration in DSS-treated *Rbbp9*^*-/-*^ mouse colons was abolished upon UPA treatment (Figure 9*H*). These data indicate that inhibiting the JAK/STAT1 axis reverses epithelial cell apoptosis, alleviating inflammatory phenotypes in *Rbbp9*^*-/-*^ mice.

## Discussion

In this study, we investigated the critical role of RBBP9 in the development of intestinal inflammation and cancer using knockout mouse models and human bioinformatics analyses. Although RBBP9 is expressed in various human cancer cells, its role in cancer development remains poorly understood. Given its ability to bind to RB, RBBP9 was initially reported to regulate RB/E2F cell cycle activity.^17^ More recently, RBBP9 was identified as a metabolic serine hydrolase that promotes cancer cell survival by undermining the anti-proliferative function of TGF-β in a pancreatic cancer model.^15^ Furthermore, a recent study using genome-wide small interfering RNA (siRNA) screening identified RBBP9 as a synthetic lethal gene essential for tumor cells deficient in Fanconi anemia (FA) proteins in a head-and-neck squamous cell carcinoma (HNSCC) model.^14^ Accordingly, the United States Food and Drug Administration-approved RBBP9-targeting drug emetine has been shown to effectively kill FA-deficient HNSCC cells. These pioneering studies elucidated the crucial role of RBBP9 in cancer development, primarily using *in vitro* biochemical approaches.

However, the context-dependent roles of this molecule in physiological and pathological states have long been elusive, owing to the lack of a suitable genetic animal model. In addition, prior to this paper, no previous study has explored the role of RBBP9 in intestinal inflammation and cancer. In this regard, we recently developed an RBBP9-knockout mouse model and discovered the non-cell-autonomous function of RBBP9 in regulating inflammation in pulmonary eosinophilia induced by the intestinal nematode *Strongyloides venezuelensis*, which migrates into the lungs.^18^ In that study, we demonstrated that RBBP9 acts as a DAMP to induce pulmonary fibroblasts to produce IL33, suppressing pulmonary inflammation.^18^ In contrast to pulmonary eosinophilic inflammation, in which fibroblasts are the main target of RBBP9, we report a previously uncharacterized cell-autonomous function of RBBP9 in directly regulating the JAK/STAT1 signaling pathway during inflammation and inflammation-induced tumorigenesis in the intestines. We did not observe changes in IL33 levels in the colitis of RBBP9-deficient intestines. This discrepancy was likely attributable to context-dependent differences in the inflammatory nature of the two inflammatory models. Nematode-induced pulmonary inflammation is an allergic inflammation wherein IL33 and ILC2 play crucial roles. In contrast, the insufficient barrier function of IECs and microbial invasion increase the production of pro-inflammatory cytokines, including TNF-α, IFN-γ, and IL6, during intestinal inflammation in murine DSS-induced colitis and human UC and CAC. These observations highlight the importance of utilizing specific *in vivo* models to better understand the role of the featured molecules in complex and context-specific inflammatory responses.

Although the activation of Wnt/β-catenin signaling is a hallmark of sCRC development, mutations in this pathway are less frequently observed in UC or CAC, suggesting that the growth signaling pathway involved in inflammatory conditions differs from that in sCRC.^19^, ^20^, ^21^, ^22^, ^23^, ^24^ Nuclear factor-κB (NF-κB), a key transcription factor in immunological and inflammatory response regulation, induces IL6 expression in immune cells from the lamina propria, further activating STAT3 expression in IECs. This activation promotes tumorigenesis by facilitating IEC proliferation and inhibiting apoptosis and other pro-tumorigenic pathways.^24^^,^^31^^,^^32^ Thus, the deletion of IL6 reduces tumorigenesis in the AOM-DSS mouse model. By mediating IL6/STAT3 activation, mTORC signaling promotes a regenerative process after injury in the intestines^33^; moreover, strong evidence links mTORC1 hyperactivation with UC and CAC.^34^, ^35^, ^36^ These findings support the concept that the increased activity of both mTORC1 and IL6/JAK/STAT3 signaling enhances tumor formation in an inflammation-dependent AOM/DSS model in RBBP9-deficient mice. In contrast, the *Apc*^*Min/+*^ mouse model, in which the development of intestinal tumors depends primarily on genetic alterations in epithelial cells rather than inflammation, showed the impaired occurrence and growth of intestinal tumors due to the loss of RBBP9. This is consistent with previous reports demonstrating the pro-tumor function of RBBP9 by inhibiting the cell cycle regulatory protein RB or suppressing the TGF-β pathway mediated by its serine hydrolase activity.^15^^,^^17^ However, RNA-seq data showed the enrichment of gene signatures related to IFN signaling, even under non-inflammatory conditions, albeit only at the transcriptomic level, suggesting that RBBP9 loss renders cells more sensitive to IFN signaling. Consequently, the addition of inflammatory stimuli enhanced colon tumor development more in *Apc*^*Min/+*^;*Rbbp9*^*-/-*^ mice than in *Apc*^*Min/+*^ mice, accompanied by the presence of stronger inflammation-mediated proliferative activity. These data suggest that the loss of RBBP9 enhances inflammation and inflammation-mediated proliferation in response to inflammatory stimuli, thereby overcoming the reduction in spontaneous tumorigenesis observed in *Apc*^*Min/+*^;*Rbbp9*^*-/-*^ mice.

In this study, we identified RBBP9 as a critical regulator of the JAK/STAT1 pathway that suppresses STAT1 activation. Our *in vitro* kinase assay revealed that RBBP9 directly suppresses STAT1 phosphorylation. Furthermore, results from experiments using emetine suggested that this inhibitory effect on STAT1 phosphorylation was mediated by the serine hydrolase activity of RBBP9. A recent report combining hydrolase substrate traps with the direct identification of Dap conjugates revealed that RBBP9 is an aminopeptidase with a preference for removing aromatic amino acids in mammalian cells.^37^ However, the precise C-terminal region of RBBP9 substrates remains elusive and requires identification in future studies.

STAT1 is an adapter protein that facilitates signaling from both IFN-α and IFN-γ to activate IFN-responsive genes. Notably, this signaling pathway is cell type-dependent: the enhanced activation of JAK/STAT1 signaling in epithelial cells results in their death, whereas STAT1 phosphorylation activates immune cells, including macrophages. This indicates that the impact of RBBP9 loss differs between epithelial and immune cells. In the intestine, IEC death causes barrier dysfunction, allowing microbial translocation from the lumen to the lamina propria. Activated macrophages stimulated by microbiota recruit CD8^+^ T cells, which produce IFN-γ, inducing IEC apoptosis and further barrier dysfunction. This process creates a positive feedback loop enhancing inflammation. These findings highlight the importance of strictly regulating the JAK/STAT1 pathway, as any disruption can easily lead to enhanced inflammation through the activation of the inflammatory circuit. They also identified RBBP9 as a fine tuner of intestinal inflammation, which is consistent with our human data showing decreased RBBP9 expression in UC and CAC samples. The simultaneous knockout of STAT1 reversed the increased inflammatory phenotypes in *Rbbp9*^*-/-*^ mice, suggesting that STAT1 could be a viable target for the treatment of UC and CAC, especially in cases of low RBBP9 expression. Among the approved JAK inhibitors, UPA showed high specificity for JAK1 and efficacy in inducing and maintaining remission in patients with UC.^12^ Hence, upadacitinib may hold promise for patients with UC, particularly for those with low RBBP9 expression and an inadequate response to other UC therapies.

In summary, our results deepen our understanding of how RBBP9 mediates the regulation of the JAK/STAT1 signaling pathway during intestinal inflammation and inflammation-induced tumorigenesis by directly suppressing STAT1 phosphorylation. These findings offer potential avenues for the treatment of UC and CAC.

## Methods

All authors had access to all the data and have reviewed and approved the final manuscript.

### Human Samples

Paraffin-embedded tissue sections of UC, CAC, and sCRC were obtained from surgically resected samples from 40 patients with UC, 40 patients with CAC, and 35 patients with sCRC. The Ethics Committee of Kyoto University and Hyogo Medical University approved the use of patient samples for this experiment without requiring written informed consent. Informed consent was obtained in the form of opt-out on the website. The study protocol (R3561) was approved by the Ehics Committee of Kyoto University Hospital.

### Bioinformatics Analysis

Raw gene expression data of human UC patient dataset (GSE9452, GSE38713, and GSE59071) were directly accessed through the Gene Expression Omnibus (GEO) website (National Center for Biotechnology Information). Heat-map representation of gene expression was generated using Morpheus (https://software.broadinstitute.org/morpheus/). Normalized count data from mouse RNA-seq data and human UC patient datasets were used for gene set enrichment analysis (GSEA) and differentially expressed genes were determined using TCC (Tag Count Comparison) with a false discovery rate cutoff value <0.1. GSEA was performed using GSEA software (http://www.gsea-msigdb.org/gsea/index.jsp).

### Mice

*Rbbp9*-deficient (*Rbbp9*^-/-^) mice were developed as previously described^18^^,^^38^ (Hyogo Medical University). *Stat1*-deficient (*Stat1*^-/-^) mice were kindly provided by Dr Tetsuji Naka and Dr Minoru Fujita (Iwate Medical University).^38^ C57BL/6 mice were purchased from Japan SLC, Inc. *Apc*^*Min/+*^ mice were purchased from The Jackson Laboratory (Stock no. 002020; The Jackson Laboratory). To generate DSS-induced colitis, male mice aged 8 weeks were administered 2.5% DSS in the drinking water for 5 days, followed by regular drinking water for 2 days, and were then sacrificed on day 7. To assess intestinal permeability, WT and *Rbbp9*^*-/-*^ mice were treated with 2.5% DSS or normal water for 5 days, followed by regular drinking water for 1 day. After a 12-hour fast, mice were orally administered FITC-dextran (600 mg/kg; TDB-FD4-1, Tdb Labs), and serum FITC levels were measured 3 hours later. For the DSS treatment on *Apc*^*Min/+*^ and *Apc* ^*Min/+*^*;Rbbp9*^*-/-*^ mice, 11-week-old mice were treated with 2% DSS for 5 days and were sacrificed at 15 weeks of age. For the treatment experiment with the JAK inhibitor, mice were orally administered UPA (20 mg/kg/day) or vehicle from day 1 to day 6 during the DSS treatment.^39^ To generate AOM/DSS colorectal tumor, male mice aged 8 weeks were intraperitoneally injected with AOM (12 mg/kg). Two days later, 2% DSS was administered in the drinking water for 5 days following by regular drinking water for 16 days. This cycle was repeated twice with 2% DSS, and mice were sacrificed at the end of the courses. For the antibiotic treatment experiment, 8-week-old WT and *Rbbp9*^*-/-*^ mice were administrated a combination of ampicillin (1 g/L; Sigma-Aldrich, A9393), neomycin (1 g/L; FUJIFILM Wako, 146-08871), metronidazole (1 g/L; Sigma-Aldrich, M3761), and vancomycin (0.5 g/L; FUJIFILM Wako, 222-01303) in drinking water for 2 weeks,^3^ followed by the treatment with 2% DSS for 5 days. Body weight and survival were monitored daily until sacrifice. All mouse strains were generated in a C57BL/6 background and were born and maintained under pathogen-free conditions. Animal handling and experimental procedures conformed to institutional guidelines and were approved by the animal research committee of Kyoto University and performed in accordance with Japanese government regulations. Male mice were used in all experiments.

### Cell and Organoid Culture

MC38 and MODE-K cells were cultured in Dulbecco’s modified Eagle’s medium (DMEM) with 10% fetal bovine serum (FBS) and 1% penicillin-streptomycin.^40^^,^^41^ Normal and tumor organoids from mouse small intestines were generated and cultured as previously described with some modification.^7^^,^^42^ Briefly, small intestines were collected, washed with cold phosphate buffered saline (PBS) without magnesium chloride and calcium (PBS−/−) opened longitudinally, cut into 5-mm fragments, and then washed several times with cold PBS−/−. Cut tissue fragments were incubated with PBS−/− EDTA (5 mM) containing 10% FBS for 30 minutes at 4 °C. Intestinal epithelial cells were then mechanically separated from the connective tissue by rigorous shaking, and then filtered through a 100-μm mesh into a 50-mL conical tube to remove tissue fragments. For small intestinal organoid culture, crypt number was counted after isolation and a total of 300 crypts were mixed with 50 μL of Matrigel and plated in 24-well plates. After polymerization of Matrigel, 500 μL of crypt culture medium (STchnologies, ST-06005) was added. For tumor organoid culture, we collected small intestinal adenoma tissues from *Apc*^*Min/+*^ and *Apc* ^*Min/+*^*;Rbbp9*^*-/-*^ mice. Isolated adenoma tissues were minced into small pieces and incubated in digestion buffer (DMEM with 10% FBS, penicillin/streptomycin, 2 mg/mL collagenase D) for 30 minutes at 37 °C. The digested adenoma fragments were washed with PBS−/− and then filtered through a 100-μm mesh into a 50-mL conical tube to remove stromal fragments. Adenoma fragments were counted after isolation and a total of 100 to 250 fragments were mixed with 50 μL of growth factor reduced-Matrigel and plated in 24-well plates. After polymerization of Matrigel, 500 μL of culture medium (Advanced DMEM/F12 containing 10 mM HEPES, 1× Glutamax, 1× B27 supplement, 50 ng/mL EGF, and 10 μM Y-27632) was added. SYTOX green staining (Thermo Fisher, S7020) was used to detect dying organoids. SYTOX green-positive organoids were quantified by fluorimetry using ImageJ.

### Collection of Bone Marrow Cells and Macrophage Differentiation

After the mice were euthanized, bone marrow (BM) cells were collected from femur and humerus.^40^ Collected BM were admixed with the Red Blood Cell Lysis Solution (Miltenyi Biotec, 130-094-183), and were cultured in DMEM supplemented with 10% FBS and 1% penicillin-streptomycin. Cells were stimulated with macrophage colony-stimulating factor (M-CSF; Miltenyi Biotec, 130-101-704) for 5 days to differentiate into macrophages (BMDM). BMDM were seeded at a density of 1 × 10^6^/well in a 6-well plate, and cultured in DMEM supplemented with 10% FBS, 1% penicillin-streptomycin. After stimulated with LPS (10 ng/mL) for 2 hours, BMDM were harvested.

### Histology, Immunohistochemistry, and Immunofluorescence

Tissues were fixed in 4% paraformaldehyde in PBS, dehydrated in 70% ethanol, and embedded in paraffin. Paraffin-embedded tissues were cut into 4-μm sections. Hematoxylin and eosin (H&E) staining was performed with standard protocols. For immunohistochemistry (IHC) analysis, sections were de-paraffinized and rehydrated, followed by boiling in a microwave for 15 minutes in citrate buffer (pH 6.0) or EDTA buffer (pH 9.0) for antigen retrieval, and endogenous peroxidase was quenched in 3% H_2_O_2_ in methanol for 10 minutes at room temperature. Blocking was performed by incubating sections with a 10% bovine serum albumin (FUJIFILM Wako, 9048-46-8;). Sections were incubated with primary antibodies overnight at 4 °C followed by incubation with secondary antibody (1:200, Goat Anti-Rabbit IgG Antibody [Vector Laboratories, BA1000], Horse Anti-Mouse IgG Antibody [Vector Laboratories, BA-2000], Rabbit Anti-Rat IgG Antibody [Vector Laboratories, BA-4000]) for 1 hour at room temperature. Immune complexes were visualized using a VECTASTAIN Elite ABC Standard Kit (Vector Laboratories, PK-6100) and a diaminobenzidine substrate (Dako, K3468), followed by counterstaining with hematoxylin. The following primary antibodies were used: RBBP9 (1:200; Proteintech, 12230-2-AP, RRID:AB_2175265), CD45 (1:100; BD Biosciences, 550539, RRID:AB_2174426), CD8 (1:1,000: Abcam, ab209775, RRID:AB_2860566), cleaved caspase-3(Asp175)(5A1E) (1:200; Cell Signaling, 9664S, PRID:AB_2070042), CD11b (1:1,000; Abcam, ab133357, RRID:AB_2650514), pSTAT1(Y701)(58D6) (1:500; Cell Signaling, 9167S, RRID:AB_561284), Ki-67 (1:200; BioLegend, BL652402, RRID:AB_11204254), pSTAT3(Y705)(D3A7) (1:200; Cell Signaling, 9145S; RRID:AB_2491009), pS6 (1:1,000; Cell Signaling, 5364S, RRID:AB_10694233), and p4E-BP1 (1:500; Cell Signaling, 2855S, RRID:AB_560835). For immunostainings for human samples, the following primary antibodies were used: RBBP9 (1:1,000; LSBio, LS-C376437, PRID:AB_3101774) and pSTAT1(Y701)(58D6) (1:200; Cell Signaling, 9167S, RRID:AB_561284). For immunofluorescence staining, the signal was visualized using the Opal 3-Plex Manual Kit (Akoya Bioscience, NEL810001KT), which allows simultaneous detection of multiple targets in the same image. Secondary antibodies (undiluted, ImmPRESS Reagent [Vector Laboratories, MP-7401, MP-7404], EnVision+ System-HRP Labeled Polymer [Dako, K4001]). Fluorophores Opal 520, Opal 570, and Opal 690 were used, and the sections were counterstained with Hoechst (Thermo Fisher, H1399). The number of whole cells and positively stained cells in IHC images were measured using the Qupath.

### Immunoblot Assay

Cells were lysed with radioimmunoprecipitation assay (RIPA) Buffer (FUJIFILM Wako, 188-02453) with protease/phosphatase inhibitor cocktail (Cell Signaling, 5872S). Protein concentration in lysates were determined by using Protein Assay Dye Reagent Concentration (Bio-Rad, 5000006). Cell extracts were denatured, subjected to sodium dodecyl sulfate polyacrylamide gel electrophoresis (SDS-PAGE), transferred to membranes (Trans Blot Turbo, Bio-Rad, 1704156). After blocking with 2% dry skim milk, the membranes were incubated with the following antibodies overnight at 4 °C: RBBP9 (1:1,000; Proteintech, 12230-2-AP), cleaved caspase-3 (Asp175)(5A1E) (1:1,000; Cell Signaling, 9664S), pSTAT1(Y701)(58D6) (1:1,000; Cell Signaling, 9167S), STAT1(D1K9Y) (1:1,000; Cell Signaling, 14994S), β-actin(13E5) (1:10,000; Cell Signaling, 4970S), SMAD2/3(D7G7)XP(R) (1:1,000; Cell Signaling, 8685S), pSMAD2/3(D27F4) (1:1,000; Cell Signaling, 8828). The secondary antibodies were peroxidase-conjugated anti-rabbit IgG (1:2,000; Thermo Fisher Scienteific, 31458). Bands were detected with the Super Signal West Pico Plus Chemiluminescent Substrate (Thermo Fisher Scientific, 34577) or the Super Signal West Femto Maximum Sensitivity Substrate (Thermo Fisher Scientific, 34094) and recorded with an Amersham Imager 600 (GE Life Sciences).

### RNA Extraction and qRT-PCR Analysis

Total RNA from mouse colon and tumor samples was extracted using the RNeasy Mini Kit (QIAGEN). Complementary DNA (cDNA) was obtained using the ReverTra Ace qRT-PCR RT Kit (TOYOBO). RT-PCR was performed in duplicate using the SYBR Green PCR Master Mix (Roche) on a LightCycler 96 (Roche). Gene expression values for each sample were normalized to the 18S RNA. The primer sequences are listed in Table 1.

**Table 1:** qRT-PCR Primer Sets

Gene	Forward primer (5'-3')	Reverse primer (5'-3')
*Rbbp9*	AGAGCATCTGGCTGCCCTTCAT	CGAGAGCATATACCTGATGTGTC
*Ifng*	GCCACGGCACAGTCATTGA	TGCTGATGGCCTGATTGTCTT
*Tnf*	GGTGCCTATGTCTCAGCCTCTT	GCCATAGAACTGATGAGAGGGAG
*Cxcl9*	GGAGTTCGAGGAACCCTAGTG	GGGATTTGTAGTGGATCGTGC
*Il33*	CTACTGCATGAGACTCCGTTCTG	AGAATCCCGTGGATAGGCAGAG
*18s*	GTAACCCGTTGAACCCATT	CCATCCAATCGGTAGTAGCG

qRT-PCR, quantitative real-time polymerase chain reaction.

### RNA-Seq Analysis

For RNA-seq, total RNA was extracted from colon tumors induced by AOM/DSS treatment in WT or *Rbbp9*^-/-^ mice or small intestinal tumors in *Apc*^*Min/+*^ and *Apc* ^*Min/+*^*;Rbbp9*^*-/-*^ mice using the RNeasy Mini Kit (QIAGEN). RNA-seq was performed by Macrogen, Inc on the Novaseq6000 platform with 2 × 100 base pair paired-end sequencing using SMART-Seq v4 Ultra Low Input RNA kit and TruSeq RNA Sample Prep Kit v2. Adaptors and low-quality bases were trimmed from the reads using Trimmomatic (version 0.39) with default parameters. Reads were mapped to the *Mus musculus* reference genome build mm10 using STAR (version 2.7.3a) and counted by RSEM (version v1.3.3). Read count data were normalized using the iDEGES/edgeR method. Normalized count data from RNA-seq analysis were used for bioinformatics analyses.

### siRNA Transfection

MODE-K or MC-38 cell lines were reverse transfected with 20 nM of Rbbp9-specific siRNA (ON-TARGETplus Mouse Rbbp9 [26450] siRNA-SMARTpool [Horizon Discovery, L-046721-01-0005]) or non-targeting siRNA (ON-TARGETplus Non-targeting pool [Horizon Discovery, D-001810-10-05]). siRNAs (final concentration of 20 nM) were prepared by complex with Lipofectamine TM RNAiMAX transfection reagent (Thermo Fisher Scientific, 13778030) (in Opti-MEM media [Thermo Fisher Scientific, 31985062]) prior to reverse transfection of cells.

### Statistical Analyses

When the data met the normal distribution tested by the D’Agostino test, statistical significance between groups was determined using a Student *t*-test (2-tailed) unless specified otherwise in the figure legend. If the data did not meet this test, a Mann-Whitney *U* test was used. Statistical significance between groups of 3 or more was determined by a 1-way or 2-way analysis of variance (ANOVA), followed by the Turkey’s multiple comparison test. Data are presented as the mean ± standard error of the mean (SEM). Survival was measured by the Kaplan-Meier method and analyzed by log-rank (Mantel-Cox) test. Statical correlation was measured using the Pearson correlation coefficient (2-tailed, confidence interval [CI] = 95%). Statistical analysis was performed using GraphPad Prism 9. Values of *P* < .05 were considered as significantly different.
